# A High-Yield *Streptomyces* TX-TL Toolkit for Synthetic Biology and Natural Product Applications

**DOI:** 10.3791/63012

**Published:** 2021-09-10

**Authors:** Ming Toh, Kameshwari Chengan, Tanith Hanson, Paul S Freemont, Simon J Moore

**Affiliations:** 1 Centre for Synthetic Biology and Innovation, South Kensington Campus, London, UK; 2 Department of Medicine, South Kensington Campus, London, UK; 3 Section of Structural and Synthetic Biology, Department of Infectious Disease, Imperial College London; 4 Sir Alexander Fleming Building, South Kensington Campus, South Kensington, London SW7 2AZ; 5 School of Biosciences, Division of Natural Sciences, University of Kent, UK; 6 UK Dementia Research Institute Care Research and Technology Centre, Imperial College London; Hammersmith Campus, Du Cane Road London W12 0NN; 7 UK Innovation and Knowledge Centre for Synthetic Biology (SynbiCITE) and the London Biofoundry, Imperial College Translation & Innovation Hub, White City Campus 80 Wood Lane, London W12 0BZ

**Keywords:** Cell-free protein synthesis, *in vitro* transcription-translation, *Streptomyces*, synthetic biology, systems biology, cell-free systems, biosynthesis

## Abstract

*Streptomyces* spp. are a major source of clinical antibiotics and industrial chemicals. *Streptomyces venezuelae* ATCC 10712 is a fast-growing strain and a natural producer of chloramphenicol, jadomycin and pikromycin, which makes it an attractive candidate as a next-generation synthetic biology chassis. Therefore, genetic tools that accelerate the development of *S. venezuelae* ATCC 10712, as well as other *Streptomyces* spp. models, are highly desirable for natural product engineering and discovery. To this end, a dedicated *S. venezuelae* ATCC 10712 cell-free system is provided in this protocol to enable high-yield heterologous expression of high G+C (%) genes. This protocol is suitable for small scale (10-100 μL) batch reactions in either 96-well or 384-well plate format, while reactions are potentially scalable. The cell-free system is robust and can achieve high yields (~5-10 μM) for a range of recombinant proteins in a minimal setup. This work also incorporates a broad plasmid toolset for real-time measurement of mRNA and protein synthesis, as well as in-gel fluorescence staining of tagged proteins. This protocol can also be integrated with high-throughput synthetic biology workflows or bespoke studies on biosynthetic pathways or single enzymes derived from high G+C (%) genes present in Actinomycetes genomes.

## Introduction

Cell-free transcription-translation (TX-TL) systems provide an ideal prototyping platform for synthetic biology to implement rapid design-build-test-learn cycles, the conceptual engineering framework for synthetic biology^1^. In addition, there is growing interest in TX-TL systems for high-value recombinant protein production in an open-reaction environment^2^, for example, to incorporate non-standard amino acids in antibody-drug conjugates^3^. Specifically, TX-TL requires a cell-extract, plasmid or linear DNA and an energy solution to catalyse protein synthesis in batch or semi-continuous reactions. While *Escherichia coli* TX-TL is the dominant cell-free system, a number of emerging non-model TX-TL systems have attracted attention for different applications^4–8^. Key advantages of TX-TL include flexible scalability (nanolitre to litre scale)^9,10^, strong reproducibility and automated workflows^8,11,12^. In particular, automation of TX-TL permits the accelerated characterisation of genetic parts and regulatory elements^8,12,13^.

In terms of reaction set-up, TX-TL requires a primary and secondary energy source, as well as amino acids, cofactors, additives, and a template DNA sequence. Nucleotide triphosphates (NTPs) provide the primary energy source to drive initial mRNA (ATP, GTP, CTP and UTP) and protein synthesis (only ATP and GTP). To increase TX-TL yields, NTPs are regenerated through the catabolism of a secondary energy source, such as maltose^14^, maltodextrin^15^, glucose^14^, 3-phosphoglycerate (3-PGA)^16^, phosphoenolpyruvate^17^ and L-glutamate^18^. This inherent metabolic activity is surprisingly versatile, yet poorly studied, especially in emerging TX-TL systems. Each energy source has distinct properties and advantages in terms of ATP yield, chemical stability, and cost, which is an important consideration for scaled-up TX-TL reactions. So far, current protocols for *E. coli* TX-TL have reached up to 4.0 mg/mL (~157 μM) for the model green fluorescent protein (GFP) using a blend of 3-PGA (30 mM), maltodextrin (60 mM) and D-ribose (30 mM) as the secondary energy source^19^.

Recently, there has been a rising interest in studying secondary metabolite biosynthetic pathways in TX-TL systems^20–22^. Specifically, Actinobacteria are a major source of secondary metabolites including antibiotics and agricultural chemicals^23,24^. Their genomes are enriched with so-called biosynthetic gene clusters (BGCs) which encode enzymatic pathways for secondary metabolite biosynthesis. For the study of Actinobacteria genetic parts and biosynthetic pathways, a range of *Streptomyces-based* TX-TL systems have recently been developed^5,6,25,26^. These specialised *Streptomyces* TX-TL systems are potentially beneficial for the following reasons: [1] provision of a native protein folding environment for enzymes from *Streptomyces* spp.^26^; [2] access to a high G+C (%) tailored tRNA pool for optimal high G+C (%) gene expression; [3] active primary metabolism, which potentially can be hijacked for the supply of biosynthetic precursors; and [4] tailoring of enzymes, precursors or cofactors from secondary metabolism present in the native cell-extract. Hence, a high-yield *S. venezuelae* TX-TL toolkit has recently been established to harness these unique capabilities^5^.

*S. venezuelae* is an emerging host for synthetic biology with a rich history in industrial biotechnology^5,27–29^ and as a model system for studying cell division and genetic regulation in Actinobacteria^30–32^. The main type strain, *S. venezuelae* ATCC 10712, has a relatively large genome of 8.22 Mb with 72.5% G+C content (%) (Accession number: CP029197), which encodes 7377 coding sequences, 21 rRNAs, 67 tRNAs and 30 biosynthetic gene clusters^27^. In synthetic biology, *S. venezuelae* ATCC 10712 is an attractive chassis for heterologous expression of biosynthetic pathways. Unlike most other *Streptomyces* stains, it provides several key advantages including a rapid doubling time (~40 mins), an extensive range of genetic and experimental tools^5,28^, lack of mycelial clumping, and sporulation in liquid media^28,33^. Several studies have also demonstrated the use of *S. venezuelae* for heterologous production of a diverse array of secondary metabolites, including polyketides, ribosomal and non-ribosomal peptides^34–38^. These combined features make this strain an attractive microbial host for synthetic biology and metabolic engineering applications. While *S. venezuelae* is not the dominant *Streptomyces* model for heterologous gene expression, with further developments, it is primed for broader use within natural product discovery.

**Figure 1:**
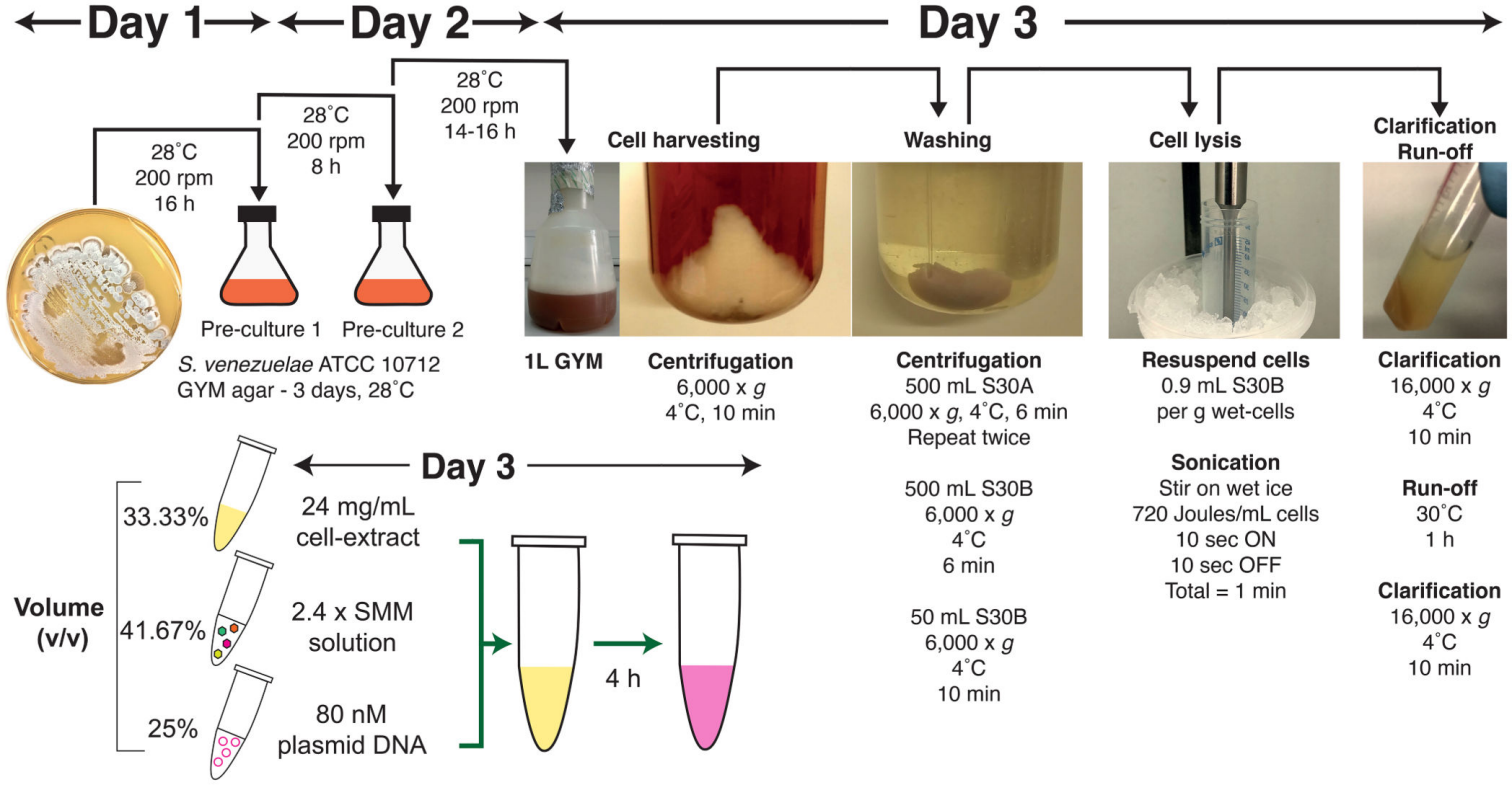
Overview of the *S. venezuelae* TX-TL protocol. A protocol summary is illustrated, including a recommended time frame of three days. The protocol is broken down into distinct stages of cell growth, cell harvest, cell wash, cell lysis by sonication, clarification, run-off reaction, energy solution (SMM) preparation, plasmid DNA preparation and the TX-TL reaction assembly. The full protocol is described in detail within the text, along with helpful guidance and practical tips.

Herein, this manuscript presents a detailed protocol (Figure 1) for a high-yield *S. venezuelae* TX-TL system, which has been updated from the original previously-published protocol^26^. In this work, the energy solution and reaction conditions have been optimised to increase protein yield up to 260 μg/mL for the mScarlet-I reporter protein in a 4 h, 10 μL batch reaction, using a standard plasmid, pTU1-A-SP44-*mScarlet-I*. This plasmid has been specifically designed to enable various methods of detecting protein expression. The protocol is also streamlined, while the energy system has been optimized to reduce the complexity and cost of setting up cell-free reactions, without compromising the yield. Along with the optimized TX-TL system, a library of genetic parts has been developed for fine-tuning gene expression as well as fluorescent tools for monitoring TX-TL in real-time, thereby creating a versatile platform for prototyping gene expression and natural product biosynthetic pathways from *Streptomyces* spp. and related Actinobacteria.

**Figure 2:**
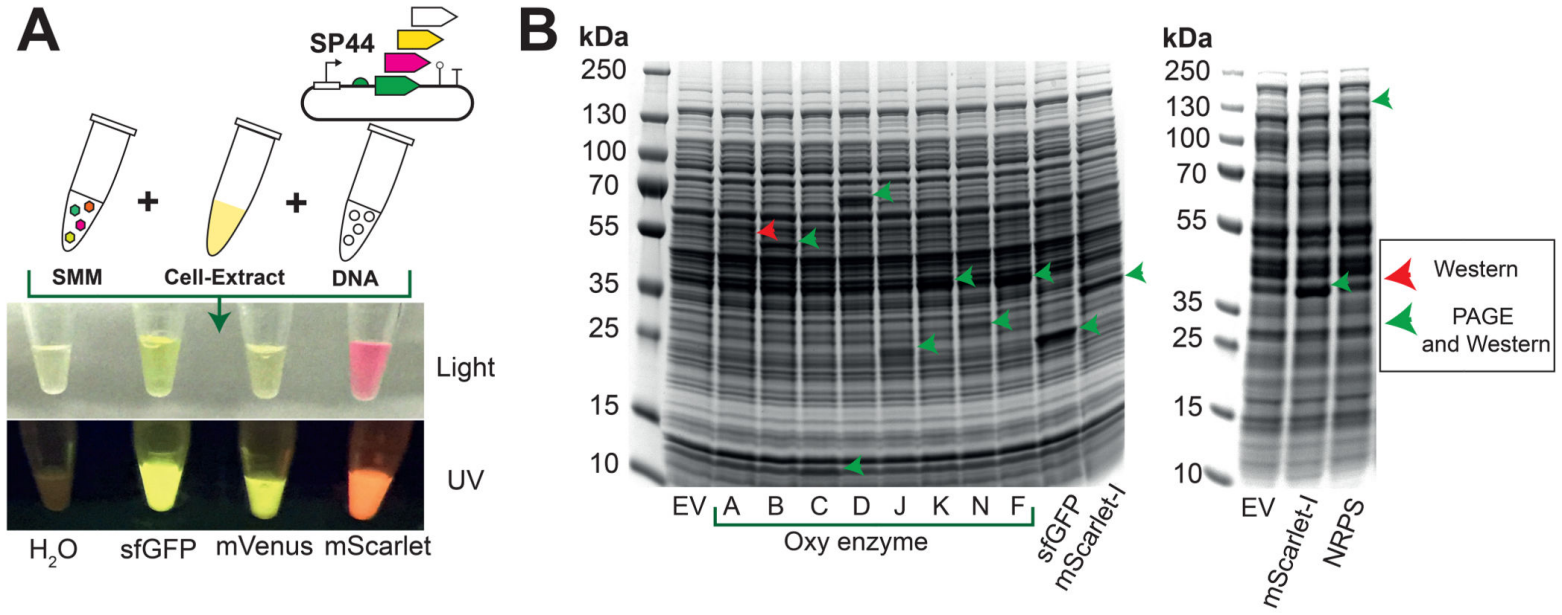
High-yield protein synthesis from high G+C (%) genes. (A) Synthesis of sfGFP, mVenus-I and mScarlet-I fluorescent proteins. (B) Synthesis of biosynthetic enzymes from *S. rimosus*. The figure is adapted with permission from ACS Synthetic Biology^5^. Please see protocol and supplementary files for reaction set-up and methodology.

In this work, the recommended standard plasmid (pTU1-A-SP44-*mScarlet-I*) can be used to establish the *S. venezuelae* TX-TL workflow in a new lab, and is available on AddGene (see Table S2). pTU1-A-SP44-*mScarlet-I* provides the user with flexibility to study other open-reading frames (ORFs). The mScarlet-I ORF is codon-optimized for *S. venezuelae* gene expression. The SP44 promoter has been shown to be a strong constitutive promoter that is highly active in both *E. coli* and *Streptomyces* spp.^39^. The plasmid has two unique restriction enzyme sites (NdeI, BamHI) to allow the sub-cloning of new ORFs in-frame with a joint C-terminal FLAG-tag and fluorescein arsenical hairpin (FlAsH) binder tag system. Alternatively, both tags can be removed with the inclusion of a stop codon after sub-cloning a new gene. With this base vector, the high-yield expression of a range of proteins has been demonstrated, namely proteins from the oxytetracycline biosynthesis pathway and an uncharacterised non-ribosomal peptide synthetase (NRPS) from *Streptomyces rimosus* (Figure 2). In terms of mRNA detection, the pTU1-A-SP44-*mScarlet-I* standard plasmid contains a dBroccoli aptamer (in the 3’-untranslated region) for detection with the 3,5-difluoro-4-hydroxybenzylidene imidazolinone (DFHBI) probe. For increased flexibility, a toolset of EcoFlex^40^ compatible MoClo parts has also been made available on AddGene, including an EcoFlex-compatible *Streptomyces* shuttle vector (pSF1C-A-RFP/pSF2C-A-RFP) and a range of pTU1-A-SP44 variant plasmids expressing superfolder green fluorescence protein (sfGFP), mScarlet-I, mVenus-I and β-glucuronidase (GUS). In particular, the pSF1C-A plasmid is derived from pAV-*gapdh*^28^ and is cured of BsaI/BsmBI sites for MoClo assembly. pSF1C-A-RFP/pSF2C-A-RFP is equivalent to pTU1-A-RFP/pTU2-A-RFP from EcoFlex^40^, but contains additional functionality for conjugation and chromosomal integration in *Streptomyces spp*. using the *phiC31* integrase system^28^.

The first stage of the protocol involves growth of the *S. venezuelae* ATCC 10712 or a closely related strain, cell harvest at mid-exponential phase, cell wash steps and equilibration in S30A and S30B buffers. This stage requires three days and the time for cell growth can be used to prepare the remaining components as described below. The harvested cells are then lysed by sonication, clarified and undergo a run-off reaction. At this final stage of preparation, the cell-extracts can be prepared for long-term storage at -80°C to minimise loss of activity. For the assembly of TX-TL reactions using this protocol, a *Streptomyces* Master Mix (SMM) is presented, with the option of a Minimal Energy Solution format (MES) that gives comparable yields. Also, it is recommended to streak a fresh culture of *S. venezuelae* ATCC 10712 from a -80°C glycerol stock onto a GYM agar plate and incubate at 28°C for at least 48-72 h until single colonies are clearly visible. Only fresh cultures should be used for the following steps.

## Protocol

### 1 Preparation of *S. venezuelae* ATCC 10712 cells

#### 1.1 Day 1 – Media/buffer preparation and overnight pre-culture

1.1.1 Prepare 1 L of sterile GYM liquid media in a 2 L baffled flask, as described in Table 1. See Table S1 for equipment/chemical/reagent sources.
1.1.2 Prepare 1 x 50 mL of sterile GYM liquid media (in 250 mL Erlenmeyer flask), as described in Table 1.
1.1.3 Prepare 100 mL 1 M HEPES, 100 mL 1 M MgCl_2_, and 500 mL 4 M NH_4_Cl solutions to make 1 L S30A and 1 L S30B wash buffers. See Table 2 for the recipes.
1.1.4 Prepare the overnight pre-culture. Pre-warm the sterile 50 mL GYM liquid media in 250 mL Erlenmeyer flask to 28 °C for 30 min.
1.1.5 Inoculate a single colony of *S. venezuelae* ATCC 10712 (or related strain) from GYM agar plate into pre-warmed 50 mL GYM liquid media and incubate at 28 °C, 200 rpm for 16 h (pre-culture 1).

#### 1.2 Day 2 – Prepare daytime pre-culture and main growth culture

1.2.1 Pre-warm 50 mL sterile GYM liquid media in 250 mL Erlenmeyer flask at 28 °C for 30 min.
1.2.2 Transfer 1 mL of overnight pre-culture into pre-warmed 50 mL GYM liquid media and incubate at 28 °C, 200 rpm for 8 h (pre-culture 2).
1.2.3 After this growth period, check the OD_600_ in a spectrophotometer using a 1:10 dilution with sterile GYM media in a 1 mL (1 cm path length) plastic cuvette. The OD_600_ should have reached at least 3-4. **Note**: If there is poor growth, it is advisable to repeat steps 1.1-1.2.2.
1.2.4 Sub-culture 0.25 mL of pre-culture 2 into 1 L of liquid GYM media in 2 L baffled flasks.
1.2.5 Leave overnight at 28 °C, 200 rpm for 14 h.

#### 1.3 Day 3 – Harvest cells

1.3.1 After the previous incubation period (14 h), record the OD_600_ of the main culture. Dilute the overnight culture 1:10 with fresh GYM media for OD_600_ measurement. The OD_600_ should have reached 3.0-4.0 at this stage.
1.3.2 If OD_600_<3.0, increase shaking speed to 250-300 rpm and grow until an OD_600_ of 3.0 is reached. Grow for no longer than an additional 2 h (16 h in total).
1.3.3 If OD_600_>3.0, transfer cultures to centrifugation containers and rapidly cool on wet ice for 30 min.
1.3.4 While waiting for the cell culture to cool on ice, prepare 4 mL of fresh 1 M dithiothreitol (DTT), S30A and S30B buffers, as described in Table 1 and keep them on ice. See Table S1 for chemical/reagent source.
1.3.5 Pre-weigh an empty 50 mL centrifuge tube and pre-chill at -20 °C.
1.3.6 Add 2 mL of 1 M DTT to 1 L of S30A buffer on ice and mix well. **Note:** Add DTT to the S30A and S30B wash buffers only before using them.
1.3.7 Centrifuge cells at 6,000 x *g*, 4 °C, 10 min and carefully discard the supernatant in a quick and single motion. **Note**: If the pellet is disturbed, maximise cell retention with residual GYM media and continue the protocol.
1.3.8 Add 500 mL of S30A buffer and resuspend the cells by shaking the centrifugation bottles vigorously until the cell clumps are homogeneously dispersed.
1.3.9 Centrifuge the cells at 6,000 x *g*, 4 °C, 6 min and carefully discard the supernatant. **Note**: The cell pellet will be firmer at this point, but some cells will remain in suspension (see Figure 1). Treat as described in 1.3.7 and retain as many cells as possible.
1.3.10 Repeat steps 1.3.8-1.3.9.
1.3.11 Add 2 mL of 1 M DTT to 1 L of S30B buffer on ice and mix well.
1.3.12 Add 500 mL of S30B buffer to the cells.
1.3.13 Repeat step 1.3.9.
1.3.14 Re-suspend the cell pellet in 10 mL S30B buffer and transfer to the pre-weighed, pre-chilled 50 mL centrifuge tube. If required, transfer residual cells with an additional 5-10 mL S30B buffer. Fill to 50 mL with S30B.
1.3.15 Centrifuge cells at 6,000 × *g*, 4 °C, 10 min and carefully discard the supernatant.
1.3.16 Repeat step 1.3.15.
1.3.17 Carefully aspirate remaining S30B supernatant with a 100-200 μL pipette.
1.3.18 Weigh the wet cell pellet. **Note:** Typical weight for 1 L of overnight GYM culture (OD_600_ = 3.0) is ~4.5 g.
1.3.19 For every 1 g of wet cells, add 0.9 mL of S30B buffer.
1.3.20 Re-suspend the cells using either a Pasteur pipette or vortex.
1.3.21 Centrifuge briefly (~10 sec) up to 500 × *g* to sediment the cells. **Note:** The protocol can be paused at this point and cells can be frozen on either liquid nitrogen or dry ice and stored at -80°C. **Safety**: Wear appropriate PPE when handling liquid nitrogen, including face shields and gloves.

### 2 Cell lysis by sonication to obtain the crude cell extract

**Note**: At this stage, the user can choose to disrupt the cells by sonication either in 1 mL fractions (**option 2.1**) or as a larger cell-suspension (5 mL) in a 50 mL tube (**option 2.2**). Both options have been detailed below to ensure reproducibility, since the final volume of the cell-suspension can change due to loss of cells between steps 1.3.1-1.3.21. A new user should attempt **option 2.1** first to establish the protocol.

#### 2.1 Cell Lysis by sonicating in 1 mL fractions

2.1.1 Using a 1 mL pipette tip (cut off end of tip to increase bore size), transfer 1 mL of cell suspension into 2 mL microcentrifuge tubes. **Note**: If cells are frozen, rapidly thaw the 50 mL tube containing the pellet with lukewarm water prior to cell lysis. Transfer the tube to wet ice as soon as the pellet has begun to defrost and chill for 10 min.
2.1.2 Place each microcentrifuge tube in a beaker of ice water, using a plastic tube rack to hold the tube for sonication. **Note**: Due to sensitivity of cell-extract to over-heating, it is critical to ensure that the tubes do not warm up to prevent protein precipitation and reduced enzymatic activity.
2.1.3 Use a sonicator probe with a 3 mm diameter tip and clean it with 70% (v/v) ethanol and double distilled water (ddH_2_O).
2.1.4 Lower the sonicator tip into the cell suspension until it is about 1 cm below the liquid surface.
2.1.5 Input the following settings into the sonicator: 20 kHz frequency, 65% amplitude, 10 sec pulse ON time, 10 sec pulses OFF time, 1 min total sonication time.
2.1.6 Run the sonication protocol. During the first two resting cycles, move the tube up/down and sideways, to ensure the cells are evenly sonicated. Record the energy input. **Safety**: Wear appropriate hearing protection during sonication. **Note**: The viscosity will decrease as cells are disrupted, and the pale cream wet cell pellet should turn into a homogenous brown fluid. The recommended energy input is 240 J per mL of well cells.
2.1.7 If the cells are only partially lysed the suspension will still appear cream coloured with viscous clumps of cells, particularly on the sides of the tube. Invert the tube 2-3 times and repeat the sonication for an additional one or two 10-sec cycles, mixing frequently, until cells are fully disrupted.

#### 2.2 Cell Lysis by sonicating a 5 mL cell suspension

2.2.1 If cells are frozen, rapidly thaw the 50 mL tube containing the pellet with lukewarm water with shaking, prior to cell lysis. Transfer the tube to wet ice as soon as the pellet as begun to defrost and chill for 10 min.
2.2.2 Briefly spin the tube at 500 x *g* to sediment the cells.
2.2.3 Place the 50 mL tube in a beaker of ice water for sonication. **Note**: Due to sensitivity of cell-extract to over-heating, it is critical to ensure that the tubes do not warm up to prevent protein precipitation and reduced enzymatic activity.
2.2.4 Use a sonicator probe with a 6 mm diameter tip and clean it with 70% (v/v) ethanol and ddH_2_O (visual schematic of 6 mm probe within Figure 1). Lower the sonicator tip into the cell suspension (~5 mL) until it is about 1 cm below the liquid surface.
2.2.5 Input the following settings into the sonicator: 20 kHz frequency, 65% amplitude, 10 sec pulse ON time, 10 sec pulses OFF time, 1 min total sonication time per mL of wet cells (5 min in total).
2.2.6 Run the sonication protocol. During the first two resting cycles, move the tube up/down and sideways, to ensure the cells are evenly sonicated. **Safety**: Wear appropriate hearing protection during sonication. **Note**: The viscosity will decrease as cells are disrupted, and the pale cream wet cell pellet should turn into a homogenous brown fluid. Record the energy input. An optimal energy input of 240 J per mL of wet cells (~1200 J in total from 5 min sonication) is recommended.
2.2.7 If some cells remain intact, follow guidance from step 2.1.7.
2.2.8 Transfer the cell-extracts into 2 mL microcentrifuge tubes.

### 3 Cell-extract clarification and run-off reaction

3.1 Centrifuge the lysed cells at 16,000 × *g* for 10 min at 4 °C to remove the cell debris.
3.2 Transfer the supernatant into 1.5 mL microcentrifuge tubes as 1 mL aliquots.
3.3 Conduct the run-off reaction for the cell-extracts. Incubate the 1.5 mL tubes containing the cell-extracts at 30 °C for 60 min on a heat block or incubator. Shaking is not required.
3.4 Centrifuge the cell-extracts at 16,000 × *g* for 10 min at 4 °C.
3.5 Pool the supernatants into a 15 mL centrifuge tube. Mix the supernatant by inverting the tube five times until homogenous, then keep it on ice. Invert gently to avoid the formation of air bubbles.
3.6 Dilute 10 μL of cell-extract 100-fold with S30B buffer and measure total protein concentration using a Bradford assay with three technical repeats (see Supplementary Material S3 for Bradford assay guidance).
3.7 If the protein concentration is between 20-25 mg/mL, transfer the cell-extracts as 100 μL aliquots into new 1.5 mL tubes, flash freeze in liquid nitrogen and store at -80 °C. **Safety**: Wear appropriate PPE when handling liquid nitrogen, including face shields and gloves.
3.8 If the protein concentration is <20 mg/mL, repeat the crude extract preparation steps to ensure high quality cell-extract and TX-TL yields comparable to the previously published work^5^.

### 4. Preparation of Plasmid DNA Template

4

4.1 Purify the pTU1-A-SP44-*mScarlet-I* plasmid (pUC19 origin) from a freshly transformed *E. coli* plasmid strain (DH10β, JM109) grown in 50 mL of LB culture (with 100 μg/mL carbenicillin) using an appropriate plasmid DNA purification kit as per manufacturer’s instructions.
4.2 Elute the plasmid in 2 x 300 μL of nuclease-free water and combine fractions.
4.3 Add 0.1 volumes (66 μL) of 3 M sodium acetate (pH 5.2).
4.4 Add 0.7 volumes (462 μL) of isopropanol.
4.5 Incubate the DNA at -20 °C for 30 min.
4.6 Centrifuge at 16,000 × *g* for 30 min at 4 °C and discard the supernatant.
4.7 Add 2 mL of 70% (v/v) ethanol to the DNA pellet.
4.8 Invert the tube 3-4 times to resuspend the plasmid DNA pellet.
4.9 Centrifuge at 16,000 × *g* for 5 min at 4 °C and discard the supernatant.
4.10 Repeat steps 4.7-4.9 and remove all visible liquid.
4.11 Air-dry the DNA pellet for 10-30 min or dry for 5 mins with a vacuum centrifuge.
4.12 Resuspend the dried pellet with 600 μL of nuclease-free ddH_2_O.
4.13 Measure the DNA concentration and purity using a spectrophotometer.
4.14 Prepare 50-100 μL aliquots and store at -20 °C. **Note:** High DNA concentration in the range of 500-1000 ng/μL is recommended due to the tight volume constraints of cell-free reactions. Dilute the plasmid DNA stock to 80 nM. 168 ng/μL pTU1-A-SP44-*mScarlet-I* plasmid is equivalent to 80 nM.

### 5 Preparation of the *Streptomyces* Master Mix (SMM) solution

#### 5.1 Amino acid solution

5.1.1 Use the RTS sampler kit to avoid manual errors and reduce preparation time, following the manufacturer’s instructions, as provided online (www.biotechrabbit.com).
5.1.2 Dilute the 20 × amino acid stock solution using ddH_2_O to a final concentration of 6 mM (5 mM L-Leu).
5.1.3 Further dilute to 2.4 mM (2 mM L-Leu) within the **2.4 × SMM** solution (see Table 3). The final concentration in the TX-TL reaction is 1 mM 19 × amino acids and 0.83 mM L-Leu.

#### 5.2 Energy solution and additives

5.2.1 Prepare the other components within the **2.4 × SMM** solution following the recipe described in Table 3.
5.2.2 Alternatively, prepare a **2.4 × MES**, following the recipe described in Table 3.

### General guidance

1. Store stocks for 1 M Mg-glutamate, 4 M K-glutamate, 40% (w/v) PEG 6000, 1.11 g/mL PVSA at room temperature. All other stocks are stable at -80 °C. Minimize the number of freeze-thaw cycles to avoid chemical degradation.
2. For preparation of energy solution stocks (see Table 3) such as 3-PGA (requires pH adjustment), follow the guidance provided in the *E. coli* TX-TL protocol^41^. All components are fully soluble in ddH_2_O and stored as aliquots in the -80 °C freezer.
3. Defrost individual stocks or **SMM** solution on ice. Heat the amino acids stock at 42 °C with vortexing for ~15-30 mins to solubilize all amino acids.
4. Keep all solutions on ice after preparation.
5. Some amino acids (L-Cys, L-Tyr, L-Leu) precipitate on ice. While minimizing rest time, leave this solution at room temperature and use a vortex to dissolve.
6. Add the calculated volumes (Table 3) of stock solutions and water and mix well using a vortex.
7. Aliquot the energy solution as 20-100 μL aliquots per tube or as desired, on ice and store at -80 °C until further use.
8. **Optional:** Conduct a TX-TL assay with the newly made **SMM** solution in comparison to the **MES** solution using 20 nM pTU1-A-SP44-*mScarlet-I* plasmid.

### 6 Setting up a standard *S. venezuelae* TX-TL reaction

6.1 Thaw the cell-extract, **SMM** (or **MES**) solution and plasmid DNA on ice. Pre-chill a 384-well plate at -20 °C.
6.2 Set up TX-TL reactions where 25% of the volume is plasmid DNA, 33.33% is cell-extract, and 41.67% is **SMM** solution and keep them on ice to avoid start time bias. A standard TX-TL template has been provided (Table 4) to calculate the volume of reagents needed based on the number of reactions. The standard volume for a 33 μL reaction is as follows: 11 μL cell-extract, 13.75 μL **SMM** and 8.25 μL plasmid DNA.
6.3 Gently vortex the mixture for ~5 sec at a low-speed setting to ensure the solution is homogenous. Avoid foaming/bubble formation.
6.4 Transfer 10 μL aliquots into three wells of a 384-well plate as a technical triplicate without introducing air bubbles. Seal the plate with a transparent cover and spin at 400 × *g* for 5 sec.
6.5 Incubate the reaction at 28 °C either in an incubator (for end-point readings) or a plate-reader. Shaking is not required. Reactions typically require 3-4 hours to reach completion. See supplementary material S3 for guidance on platereader and mScarlet-I standard measurements.

## Representative Results

**Figure 3:**
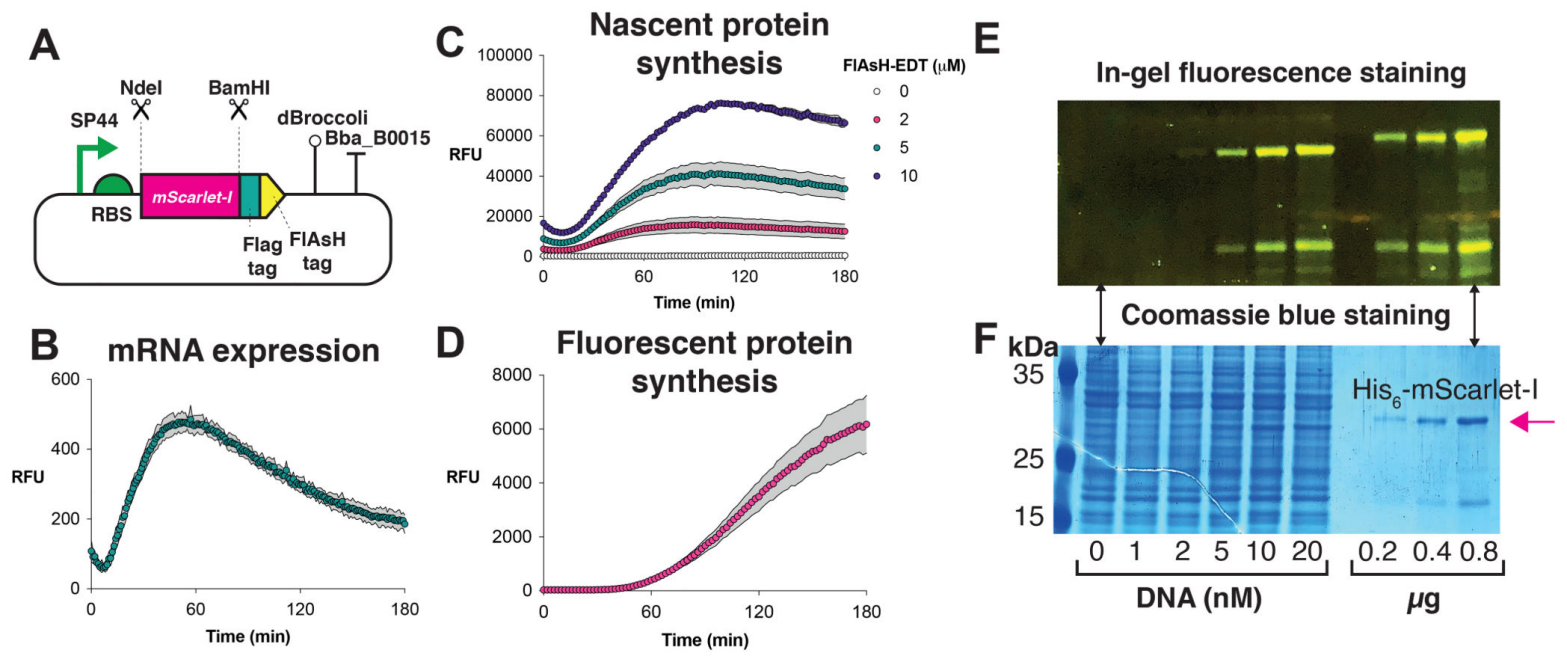
Measurement of TX-TL five-ways with the pTU1-A-SP44-*mScarlet-I* plasmid. **(**A) Plasmid design including the following features: SP44 is a strong constitutive promoter active in *Streptomyces* spp. and *E. coli;* pET-RBS is derived from the pET expression plasmids and is highly active in both *Streptomyces* spp. and *E. coli* ^5,40^; *Streptomyces* codon-optimised *mScarlet-I* gene, which encodes a rapid folding red-fluorescent protein derivative^44^; C-terminal FLAG-tag for affinity chromatography purification or Western blotting detection; C-terminal FlAsH tag for fluorescent labelling for in-gel staining or real-time measurement of nascent protein synthesis; dBroccoli aptamer for real-time mRNA measurement using the DFHBI probe; Bba_B0015 transcription terminator, which have been shown to be highly efficient in *S. venezuelae* ATCC 10712^5^; ampicillin resistance marker; and pUC19 origin of replication. (B) Real-time mRNA expression, detected with the dBroccoli aptamer and the DFHBI probe (excitation 483-14 nm, emission 530-30 nm). (C) Real-time nascent protein synthesis detection with FlAsH-EDT_2_ fluorescent probe (excitation 500-10 nm, emission 535-10 nm). (D) Real-time fluorescence measurement of mScarlet-I synthesis (excitation 565-10 nm, emission 600-10 nm). (E) In-gel staining with the FlAsH-EDT_2_ fluorescent probe. (F) Coomassie blue staining of total TX-TL proteins with purified His_6_-mScarlet-I standard for comparison. Reactions were run under the conditions described in the protocol with 40 nM of plasmid DNA template. All fluorescence data is represented as relative fluorescence units (RFU) and error bars (standard deviation of three technical repeats) are represented within a grey shaded area.

This detailed protocol is provided as an example to help the user establish a *Streptomyces* TX-TL system, based on the *S. venezuelae* ATCC 10712 model strain (Figure 1). The user may seek to study other *Streptomyces* strains, however, the growth/harvesting stages of other strains with longer doubling times or growth preferences, will need to be custom optimized to achieve peak results. For the representative result, the mScarlet-I fluorescent protein from the pTU1-A-SP44-*mScarlet-I* standard plasmid (Figure 2 and Figure 3) was optimised to provide high-yield expression in *S. venezuelae* TX-TL with a range of detection methods (SDS-PAGE, fluorescence). In addition, this standard plasmid was modified to demonstrate the synthesis of a range of secondary metabolite enzymes from *S. rimosus* (Figure 2)^5^. Finally, a potential workflow for scaled-up natural product biosynthesis is shown as a schematic workflow using a model pathway for the early-stages of haem biosynthesis. The workflow is potentially adaptable to other secondary metabolite biosynthetic pathways. As a guideline, this protocol should provide a minimum yield of 2.8 μM for sfGFP and 3.5 μM for mScarlet-I/mVenus from the expression plasmids provided on AddGene. These figures allow for typical batch variation (up to 28%) observed in previous data^5^, although yields greater than 10 μM mScarlet-I have been achieved with optimal batches (unpublished data).

### Measuring *S. venezuelae* TX-TL of mScarlet-I gene using five distinct methods

The expression of pTU1-A-SP44-*mScarlet-I* standard plasmid is shown, with the measurement of mScarlet-I expression using five different methods: 1. real-time fluorescence measurement of mRNA using the dBroccoli aptamer, 2. real-time fluorescence measurement of immature mScarlet-I protein using the FlAsH tag system, 3. real-time fluorescence measurement of mature mScarlet-I protein, 4. in-gel fluorescence staining of mScarlet-I using FlAsH tag, and 5. Coomassie blue staining of total cell-free proteins. For this data, the reactions were set-up in 2 mL microcentrifuge tubes as 33 μL reaction (for end-point samples), or as a 10 μL technical triplicate in 384-well plates in an Omega (BMG) platereader. A triple tagged (N-terminal His6, C-terminal Flag and C-terminal FlAsH) mScarlet-I protein was separately purified to create a calibration standard for measurements, using the pET15b-*mScarlet-I* plasmid, which is described further in Supplementary Material S3. The data for these experiments is shown in Figure 3. Further details of the in-gel fluorescence staining method is available in Supplementary Material S4.

### *S. venezuelae* TX-TL of early-stage haem biosynthesis

**Figure 4:**
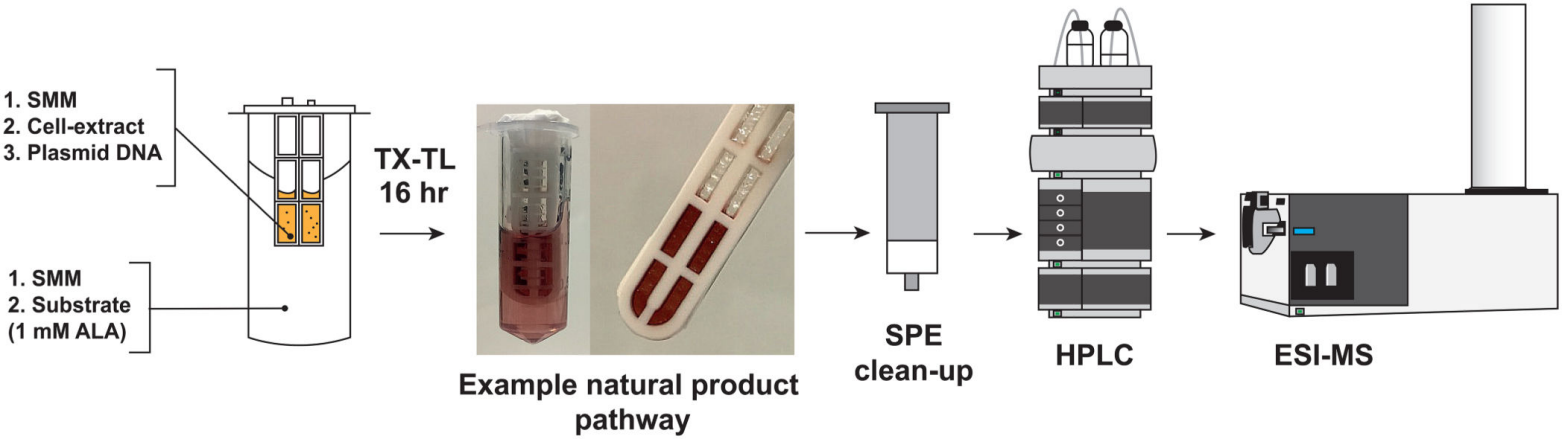
Schematic workflow for the *S. venezuelae* TX-TL semi-continuous reaction. An example workflow for natural product TX-TL, using the early-stage haem biosynthetic operon, and downstream analysis by HPLC-MS. Reactions and analysis are detailed in the supplementary. The figure is adapted with permission from ACS Synthetic Biology^5^.

To serve as a model natural product biosynthetic pathway, the ‘one-pot’ biosynthesis of uroporphyrinogen III (uro’gen III) was performed using the *pTU1-A-SP44-hemC-hemD/cysG^A^-hemB* expression plasmid^5^. This model biosynthetic pathway was chosen since uro’gen III is highly oxygen sensitive and rapidly oxidizes (loss of 6-electrons) to uroporphyrin III, which displays strong red fluorescence. This enables the reaction to be easily detected in real-time using fluorescence measurements and/or HPLC-MS (Figure 4), as previously described^5^. In addition, these reactions were studied using either a batch or semi-continuous method. A semi-continuous reaction is a strategy that uses a micro-dialysis device^42,43^ that provides additional energy (NTPs, secondary energy source) and amino acids in order to prolong the reaction time period and increase protein synthesis yields. Here, the semi-continuous method is used to scale-up the haem model reaction and separate the TX-TL proteins from the reaction product to facilitate purification and analysis by HPLC-MS. Further details of methods are available in Supplementary Material S5 or for data, please see previous work^5^. Semi-continuous cell-free reactions are also described in earlier work^42,43^. The example schematic workflow demonstrated here (Figure 4) is potentially adaptable to other natural product biosynthetic pathways.

## Discussion

In this manuscript, a high-yield *S. venezuelae* TX-TL protocol has been described with detailed steps that are straightforward to conduct, for both experienced and new users of TX-TL systems. Several features from existing *Streptomyces*^45^ and *E. coli* TX-TL^41^ protocols have been removed to establish a minimal, yet high-yield protocol for *S. venezuelae* TX-TL^5,26^. The workflow recommended here is to ensure that *S. venezuelae* is growing rapidly in the chosen rich medium, to be able to inoculate the final culture in the evening. This allows cell harvest at peak growth the following morning and allows the user to harvest and prepare the active cell-extract on the same day. By following this streamlined protocol, it is expected that a single researcher can complete the protocol conveniently in a three-day framework. A complementary plasmid toolkit has also been provided for the *S. venezuelae* TX-TL system, including a strong expression plasmid system (pTU1-A-SP44-*mScarlet-I*) which provides broad functionality for mRNA/protein analysis. This standard plasmid is powered by the constitutive SP44 promoter that is highly active in a range of *Streptomyces spp*., as well as in *E. coli*^39^. To demonstrate the initial potential of the *S. venezuelae* TX-TL toolkit, the representative results show the high-yield synthesis of a range of fluorescent proteins, secondary metabolite enzymes and the biosynthesis of a model natural product pathway (from haem biosynthesis).

Overall, the protocol contains a detailed description of the *S. venezuelae* TX-TL system, as well as practical tips for preparing the three essential components of the TX-TL reaction: (1) cell-extract, (2) *Streptomyces* Master Mix (SMM) solution and (3) plasmid DNA. This protocol does not require specialized equipment and only requires routine microbiology and biochemistry skills; hence it is accessible to most labs. The protocol is suited for both small-scale (10-100 μL) and larger-scale reactions (~2.5 mL), although some optimisation of reaction size/aeration may influence protein yield. The recommended reaction volume is 33 μL in 2 mL tube, or 10 μL in a 384-well plate. The crude extract takes five days to make by a single person starting from a glycerol stock, and each litre (L) of culture yields at least 5 mL of cell-extract (equivalent to ~1500 × 10 μL TX-TL reactions) – this is a conservative estimate and accounts for sample loss during wash steps and cell-extract clarification. Each individual stage of the protocol is independent and can be optimised by the

user to meet their own needs. A major limitation for all cell-free systems is batch variation^46,47^. Generic factors include pipetting error, user experience, media batch variation and equipment differences. We specifically introduce a master mix to minimise pipetting error and provide detailed instructions that cover media and equipment use. To date, the protocol is reproducible by a range of users in at least five UK research groups. However, it is unknown what role biological variation contributes to cell-free batch variability. Alongside global gene expression regulation differences, genome plasticity in *Streptomyces* spp. is widely reported and a potential contributor^48^. To investigate batch variation, it is recommended to grow up to four separate 1 L cultures derived from four single colonies grown overnight. Previously up to 28% variation (in terms of standard deviation) was observed between four biological batches (4 L per batch provided ~20 mL cell-extract)^5^. Based on this data, a reasonable minimal target for a new user is 2.8 μM for sfGFP and 3.5 μM mScarlet-I/mVenus-I using the plasmids that are available on AddGene – these targets are 30% less than the average observed in previous data. If downstream HPLC-MS analysis is desired, the PEG 6000 can be removed from the master mixes, although expect a decrease in the overall TX-TL yield by up to 50%.

In terms of the potential of specialized *Streptomyces* cell-free systems^5,6^, there is a growing desire to develop new wet-laboratory tools for bioprospecting applications such as natural products. The *Streptomyces* genus is steeped in the history of natural product discovery including antibiotics, herbicides, and pharmaceutical drug^49^. The increasing knowledge gained from whole-genome sequencing projects and the latest bioinformatic tools^50–52^ has revealed an unprecedented level of natural products encoded by BGCs within microbial genomes^53^. Unlocking this genetic information – which is anticipated to hold new drugs/chemicals, as well as enzymes useful to biotechnology – will require the development of new synthetic biology strategies including novel expression systems and a range of metabolic engineering tools^54^. Specialized *Streptomyces*-based TX-TL systems are advantageous to study genes and regulatory elements from Actinobacteria and related genomes for the following reasons: [1] availability of a native protein folding environment^26^, [2] access to an optimal tRNA pool for high G+C (%) gene expression, and [3] an active primary metabolism for the potential supply of biosynthetic precursors. In addition, a key advantage of cell-free systems is the high-throughput characterisation of genetic parts and gene expression, using next-generation sequencing^13^ and acoustic liquid handling robotics^8,11,12^. In summary, the *S. venezuelae* TX-TL toolkit^5^ provides a complementary tool within the field of synthetic biology for natural products. The *S. venezuelae* TX-TL toolkit will support the further development of *S. venezuelae* as a model system, as well as provide a method to engineer novel synthetic biology parts/tools and explore secondary metabolite biosynthetic pathways and enzymes.

## Supplementary Material

Supplementary file S3

Supplementary file S4

Supplementary file S5

Table S1

Table S2

## Figures and Tables

**Table 1:** Recipe for GYM bacterial growth media and GYM agar plate.

Media	Concentration (per L)	Volume	Notes
GYM agar plate	0.8 g D-glucose	200 mL	Dissolve with ddH_2_O. Adjust pH to 7.2 with 5 M NaOH before adding agar.
	0.8 g yeast extract		
	2 g malt extract		
	0.4 g CaCO_3_		
	2.4 g technical agar		
GYM media	4 g D-glucose	1L	Dissolve with distilled water and adjust pH to 7.2 with 5M NaOH.
	4 g yeast extract		
	10 g malt extract		

These components are required for routine *S. venezuelae* growth. For preparation of plasmid DNA from *E. coli*, we recommend using standard media/protocols from Sambrook *et al* (46).

**Table 2:** Reagents for preparing S30A and S30B wash buffers, adapted from Kieser *et al* ^45^

Reagent	Concentration	Amount	Additional Notes
HEPES	1 M	0.1 L	Adjust pH to 7.5 with 5M KOH
MgCl_2_	1 M	0.1 L	
NH_4_Cl	4 M	0.5 L	Takes time to dissolve (endothermic). Use a stir bar and let equilibrate to room temperature.
ddH_2_O for S30A	55.5 M	730 mL	For S30A.
ddH_2_O for S30B	55.5 M	927.5 mL	ForS30B.
DTT	1 M	5 mL	Make fresh on the day of use and keep on ice.
The above reagents are required to prepare 1L of the S30A and S30B buffers. Dissolve all components fully in ddH_2_O. Autoclave separately. We recommend using a pen to mark the side of container (Duran flask)			
**S30A buffer**			
On the day of use, add the following in order. Keep on ice.			
**Component**	**Stock concentration**	**Volume**	**Final concentration**
Deionized water	55.5 M	730 mL	
HEPES	1 M	10 mL	10 mM
MgCl_2_	1 M	10 mL	10 mM
NH_4_Cl	4 M	250 mL	1 M
DTT	1 M	2 mL	2 mM
**S30B buffer**			
On the day of use, add the following in order. Keep on ice.			
**Component**	**Stock concentration**	**Volume**	**Final concentration**
ddH_2_O for S30B	55.5 M	927.5 mL	
HEPES	1 M	50 mL	50 mM
MgCl_2_	1 M	10 mL	10 mM
NH_4_Cl	4 M	12.5 mL	50 mM
DTT	1 M	2 mL	2 mM

**Table 3:** Recipe for making the *S. venezuelae* MES and SMM solutions.

MES	Stock (mM)	2.4X solution (mM)	Volume to pipette (uL)
HEPES pH 8	2000	60	30
NTP	25	7.2	288
Amino acids	6	2.4	400
Mg-glutamate	1000	9.6	9.6
K-glutamate	4000	360	90
PEG 6000	40	2.4	60
3PGA	1400	72	51.4
ddH_2_O			71
Total volume (uL)			1000
Note: Amino acids, K-glutamate and PEG 6000 can be omitted but expect reduced activity.			

**SMM**	Stock (mM)	2.4X solution (mM)	Volume to pipette (uL)
HEPES pH 8	2000	60	30
NTP	25	2.4	96
Amino acids	6	2.4	400
Mg-glutamate	1000	9.6	9.6
K-glutamate	4000	360	90
PEG 6000	40	2.4	60
3-PGA	1400	72	51.4
G6P	1000	12	12
PVSA (mg/ml)	1000	12	12
ddH_2_O			239
Total volume (uL)			1000
Aliquot 50 mL aliquots and store at -80°C			
Troubleshooting:			
We have provided instructions for making 1 mL of MES/SMM, which minimises pipetting error and reduces freeze-thaw cycles of stocks.			
100 uL (1/10 volume) MES/SMM aliquots can be prepared to initially validate the protocol.			
If activity is low, optimisation of the Mg-glutamate and K-glutamate levels may be required between cell-extract batches. This is a guideline that existing TX-TL/CFPS protocols recommend performing.			

**Table 4:** Recipe for *S. venezuelae* TX-TL reaction.

Component	Volume (fraction)	Reactions - Equivalent to one 10 uL reaction						
		1	3	5	10	50	100	
Extract (24 mg/mL)	1/3	3.67	11	18.3	36.67	183.33	366.67	**Volume (uL)**
DNA (80 nm)	1/4	2.75	8.25	13.75	27.5	137.5	275	
MES/SMM (2.4X)	5/12	4.58	13.75	22.96	45.83	229.17	458.33	
Total volume (uL)		11	33	55	111	555	1110	

Note: 10% extra (dead volume) is added to allow for pipetting error

## Summary

This protocol details an enhanced method for synthesising high-yields of recombinant proteins from a *Streptomyces venezuelae* cell-free transcription-translation (TX-TL) system.
