# Supplementary material for: A High-Yield Streptomyces TX-TL Toolkit for Synthetic Biology and Natural Product Applications
Source: J Vis Exp. Author manuscript; Available in PMC 2023 Aug 10. (PMC7614929; doi:10.3791/63012)
Supplement: Supplementary file S3 [file EMS154240-supplement-Supplementary_file_S3.docx]

**Use of mScarlet-I as reporter protein in cell-free protein synthesis**

**1. Expression of mScarlet-I protein in *E. coli* Rosetta cells**

mScarlet-I protein was expressed using a workflow with auto-induction media, described by Levine, M. Z. *et al* (2020). Please refer to this published manuscript for details about media preparation.

1. Transform 20 µL of competent *E. coli* Rosetta (DE3) pLysS cells with 2 μL of 50 ng/μL pET15b-*mScarlet* gene and plate on LB agar with 35 μg/mL chloramphenicol and 100 μg/mL carbenicillin. Grow for 16 h at 37 °C.
2. Set up an overnight starter culture by inoculating 6 mL of auto-induction media as follows:
   1. Loopful of ~5 colonies
   2. 5.76 mL auto-induction media
   3. 0.24 mL sugar solution
   4. Antibiotics
3. Incubate at 37 °C for 16-18 h.
4. Set up the expression culture by inoculating the following:
   1. 6 mL starter culture
   2. 576 mL auto-induction media
   3. 24 mL sugar solution
   4. Antibiotics
5. Incubate at 28°C for 24 h, 200 rpm

**2. Purification of mScarlet-I protein**

1. During cell growth, prepare the following buffers.

| Buffer | Tris-HCl pH 8.0 (mM) | NaCl (mM) | Imidazole (mM) | Volume (mL) |
| --- | --- | --- | --- | --- |
| Binding buffer | 20 | 500 | 5 | 500 |
| Wash buffer I |  |  | 30 | 500 |
| Wash buffer II |  |  | 70 | 500 |
| Elute buffer |  |  | 400 | 500 |

1. Transfer the culture to 1 L centrifuge bottles and place on ice for 20 min.
2. Centrifuge at 1469 x *g* for 15 min at 4°C.
3. Discard the supernatant and re-suspend the pellet with 15 mL of binding buffer.
4. Transfer re-suspended cells into a 50 mL tube.
5. Place the 50 mL tube in a beaker of ice water for sonication. Note: Due to sensitivity of cell extract to over-heating, it is critical to ensure that the tubes do not warm up to prevent protein precipitation and reduced enzymatic activity.
6. Use a sonicator probe with a 6 mm diameter tip and clean it with 70% (v/v) ethanol and ddH_2_0. Lower the sonicator tip into the cell suspension until it is about 2 cm below the liquid surface.
7. Input the following settings into the sonicator: 20 kHz frequency, 65% amplitude, 5 sec pulse ON time, 5 sec pulse OFF time, total duration of 5 min.
8. Transfer the lysed cells to 30 mL centrifuge bottles. Take a 30 µL sample and store on ice for SDS-PAGE analysis.
9. Centrifuge the total cell fraction at 16,000 x *g* for 20 min at 4oC.
10. Resuspend the pellet in 10 mL binding buffer for SDS-PAGE analysis.
11. During centrifugation, wash an empty PD-10 column containing 3 mL IMAC resin with water.
12. Charge the column with 5 mL of 0.1 M nickel sulphate.
13. Wash the column with 10 mL ddH_2_O.
14. Equilibrate the column with 10 mL binding buffer.
15. After centrifugation in step 9, pool the supernatant and apply to the equilibrated column. Take a 30 µL sample and store on ice. Note: From this point, store each flow-through and elution fractions on ice to minimise degradation of protein.
16. Collect the flow-through.
17. Apply 50 mL of binding buffer and collect the flow-through.
18. Apply 50 mL of wash buffer I and collect the flow-through.
19. Apply 25 mL of wash buffer II and collect the flow-through.
20. Apply 5 mL of elution buffer and collect 1 mL fractions.
21. Combine the elution fractions and dialyse in a buffer containing 50 mM Tris-HCl buffer, 200 mM NaCl and 15% (v/v) glycerol overnight. Use a dialysis tubing with MWCO of 12-14 kDa.
22. For SDS-PAGE analysis of the purification fractions, transfer 30 µL of each step of the purification process into 1.5 mL microcentrifuge tubes.
23. Dilute the samples with 10 µL 4X SDS-PAGE reducing buffer and boil for 5 min.
24. Run a 12% (v/v) acrylamide gel in Tris-glycine buffer and stain with Coomassie Blue dye.
25. After dialysis, aliquot the purified protein into 0.5 mL aliquots and store at -20°C.
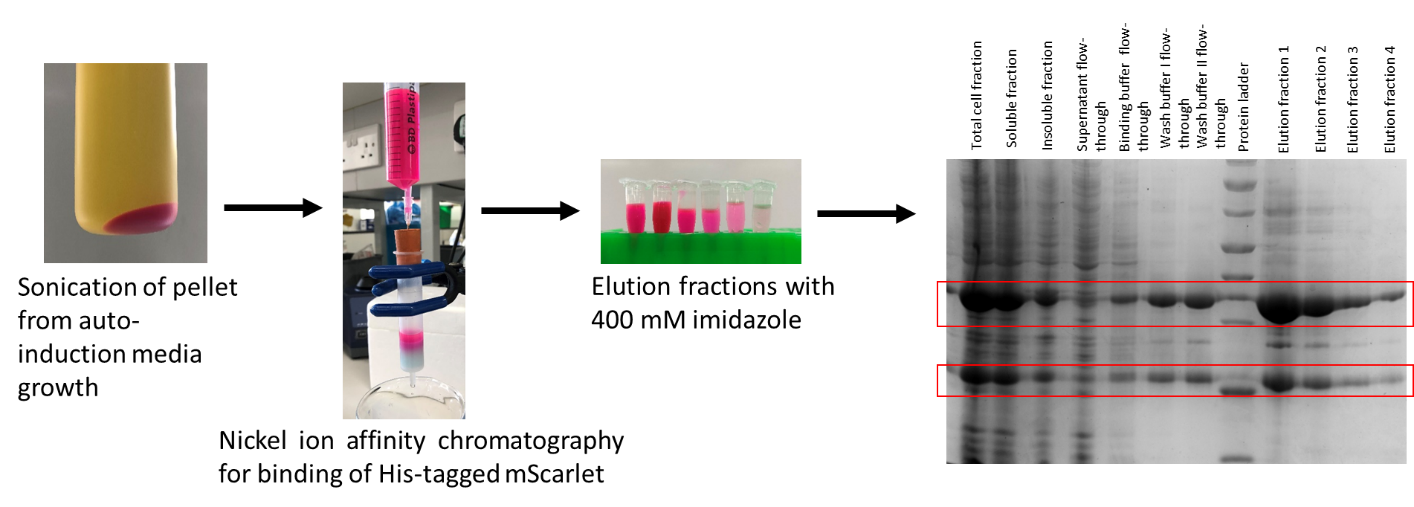


Figure 1. Example purification of mScarlet-I and SDS-PAGE gels of fractions.

**3. Estimating mScarlet-I concentration in purified and cell-free reaction samples**

Due to variability in different protein quantification assays, it is recommended to conduct the following protein assays to estimate the concentration of purified mScarlet-I with high accuracy. We recommend using mScarlet-I absorbance maxima at 569 nm to estimate protein concentration using the extinction coefficient at that wavelength, although we also verify with SDS-PAGE and a Bradford assay.

A. Bradford assay

1. Prepare 20 μL of bovine serum albumin (BSA) standards in disposable cuvettes, making dilutions between 0.2 and 0.9 mg/mL.
2. Prepare 1:50 and 1:100 dilutions of purified mScarlet-I and transfer 20 μL to a cuvette.
3. Transfer 980 μL of Bradford dye to each cuvette (20 μL of buffer as negative control). Mix well by pipetting up and down.
4. Incubate the samples at room temperature for 10 min.
5. Measure the absorbance at an optical density of 595 nm.
6. Generate a standard curve using the OD_595_ values for the BSA standards. Note: Reject the standard curve if r^2^ < 0.98 and repeat.
7. Determine the stock concentration of purified mScarlet-I using the standard curve.

Figure 2. Calibration standard curve using bovine serum albumin for Bradford assay.

B. Absorbance measurement at 569 nm

1. Prepare 1:100 dilutions of mScarlet-I in 1 mL, in technical triplicates.
2. Transfer 1 mL of each dilution to a disposable cuvette.
3. Measure the absorbance at 569 nm using an ultraviolet-visible spectrophotometer.
4. calculate the concentration of mScarlet Using the extinction coefficient with absorbance maxima at 596 nm and using the formula *Concentration in molar = Absorbance/(extinction coefficient*path length).*

C. SDS-PAGE of protein standards and mScarlet-I

1. Prepare serial dilutions of carbonic anhydrase used as the protein standard. Note: Carbonic anhydrase was chosen as protein standard for SDS-PAGE because of its comparable molecular weight to mScarlet-I protein.
2. Dilute the standards with 4x reducing buffer and boil for 5 min.
3. Load 5 μL of each protein standard and purified mScarlet-I into the wells of a 12% (v/v) acrylamide gel.
4. Run the gel in Tris-glycine buffer and stain with Coomassie Blue dye overnight.
5. Destain the gel in ddH_2_O until the background stain is minimal.
6. Take an image of the gel and measure the intensity of the bands using an image processing software such as ImageJ.
7. Estimate the concentration of purified mScarlet-I from the relative intensity of the bands corresponding to carbonic anhydrase standards.


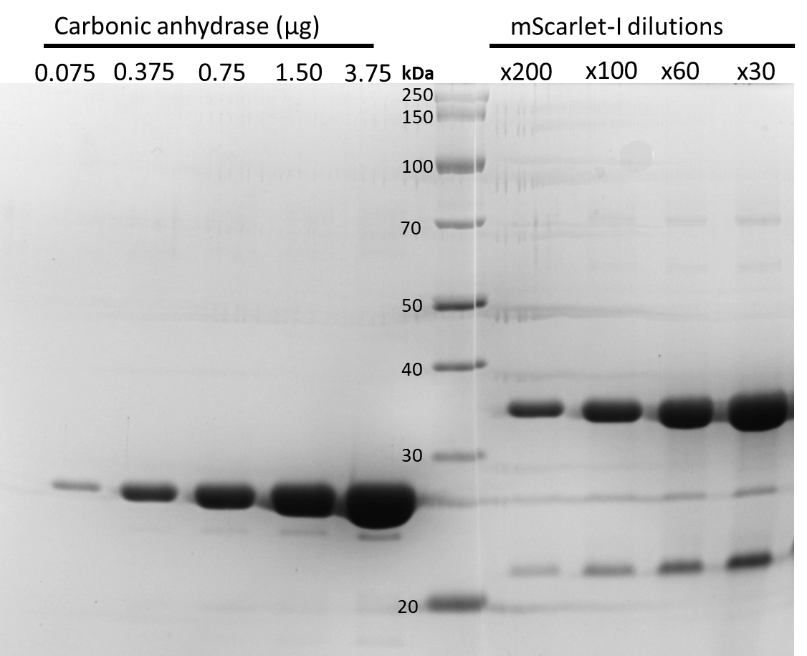


Figure 3. SDS-PAGE gel of carbonic anhydrase protein standards and mScarlet-I dilutions. Note: mScarlet-I/RFP proteins show an additional band at around ~22 kDa. We believe this is a fragment cleaved off during boiling for SDS-PAGE. In comparison, a single band/peak is observed on native PAGE or analytical size-exclusion chromatograph.

**4. Generating a calibration standard curve for estimating protein yield in cell-free protein synthesis**

1. Prepare serial dilutions of mScarlet-I (x4 final concentration) ranging from 0 μM to 10 μM.
2. Transfer 24.75 μL of mastermix into 0.2 mL PCR tube. Optional: To avoid unnecessary use of costly cell-free reagents, the mastermix can be made with crude extract and 50 mM HEPES buffer only.
3. Transfer 8.25 μL of mScarlet-I dilution to each tube, and 8.25 μL of water as negative control. Mix by pipetting.
4. Transfer the samples in 10 μL technical triplicates in a 384-well plate.
5. Seal the plate with an adhesive aluminium foil.
6. Briefly centrifuge the plate at 2,000 x *g* for 10 seconds to collect the sample at the bottom of the plate.
7. Set up the plate-reader to measure the end-point fluorescence at 28°C. Details for setting up the microplate reader are described below.
8. Use standard filters for mScarlet-I when using the Omega plate reader or monochromator settings depending on the type of microplate reader available. Use a gain of 1000.
9. Use the RFU values to generate a calibration standard curve as illustrated below.


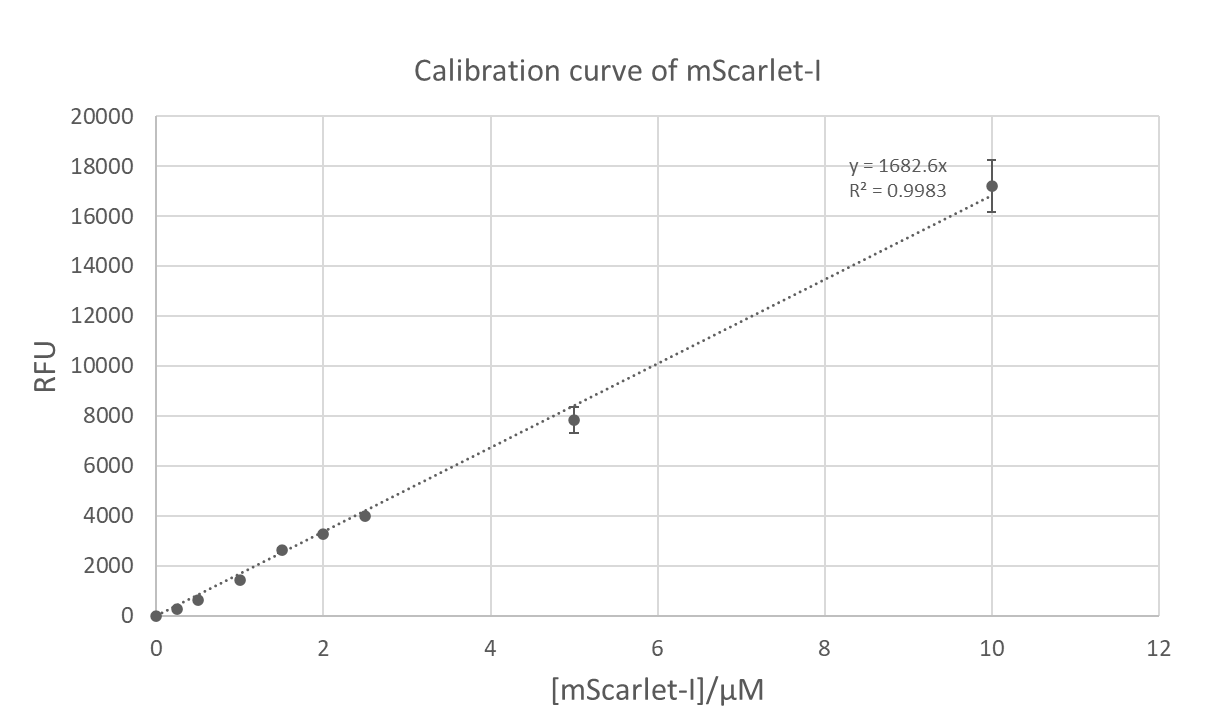


Figure 4. Calibration standard curve of purified mScarlet-I used for estimating the yield of mScarlet-I in cell-free protein synthesis.

**5. Real-time fluorescence measurement of mature mScarlet-I protein using the FLUOstar Omega microplate reader**

1. Prepare 10 μL cell-free reactions as technical triplicate repeats in a 384-well plate (black, clear flat-bottom, low-binding), keeping the plate on ice.
2. Seal the plate with an adhesive aluminium foil.
3. Briefly centrifuge the plate at 2,000 x *g* for 10 seconds to collect the sample at the bottom of the plate.
4. Set up a plate-reader to measure fluorescence at 28 °C with 5-10 seconds of shaking prior to measurements. The parameters and set-up process for “Fluorescence measurement – plate mode” on the plate reader is illustrated below. Optional: Set the number of cycles to 1 for end-point measurements.
5. Use standard filters for mScarlet-I when using the Omega plate reader or monochromator settings depending on the type of microplate reader available.
6. Measure the fluorescence of a serial dilutions on purified mScarlet-I protein, using the same microplate reader settings. Use this data to estimate the protein concentration of the cell-free reactions.

**
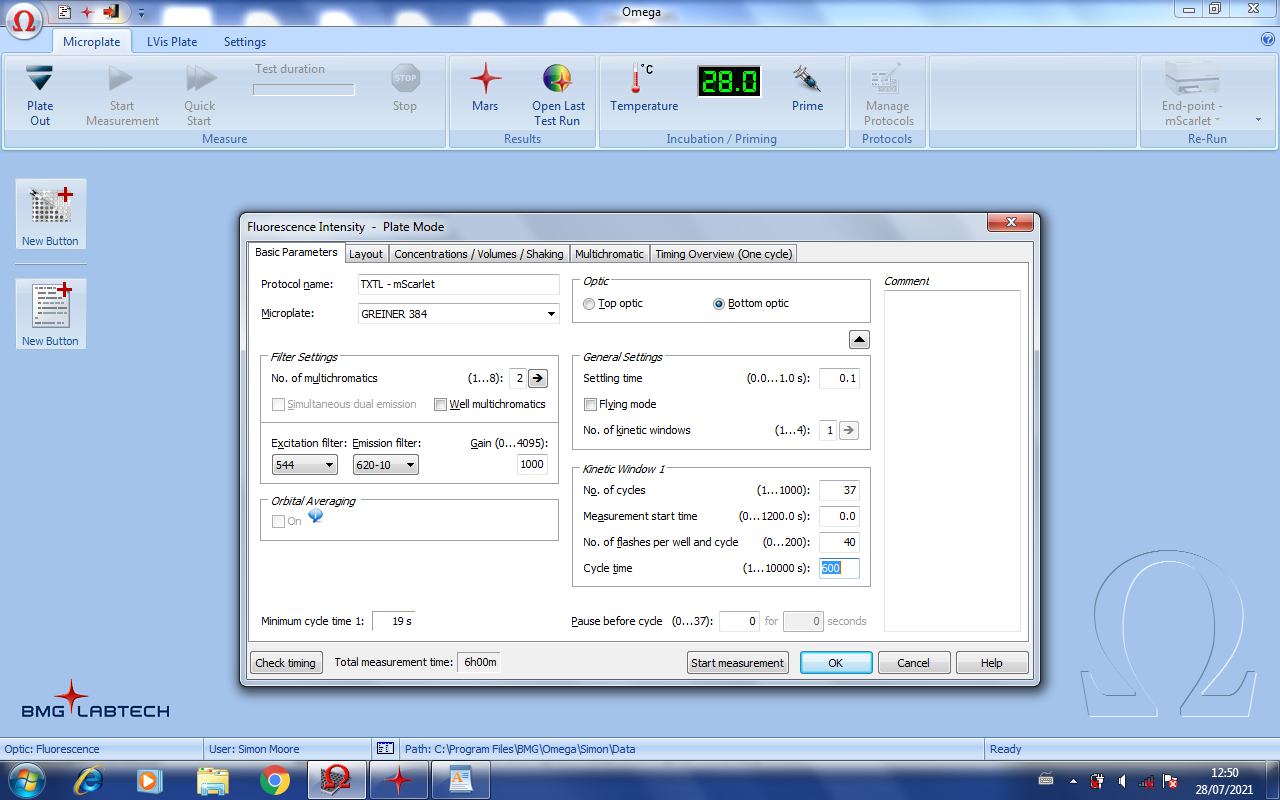
**

Figure 5. Settings used for a time-course fluorescence measurement.

**
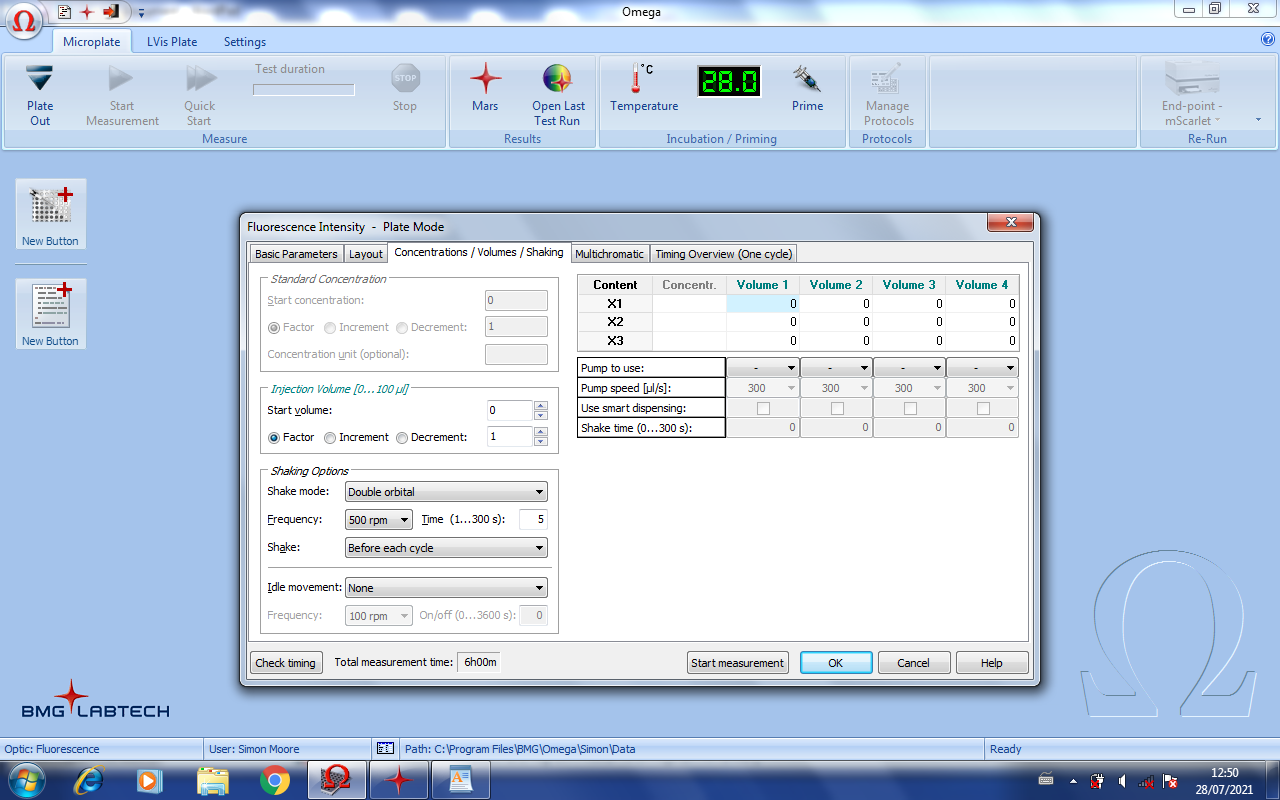
**

Figure 6. Shaking parameters used during fluorescence measurements.


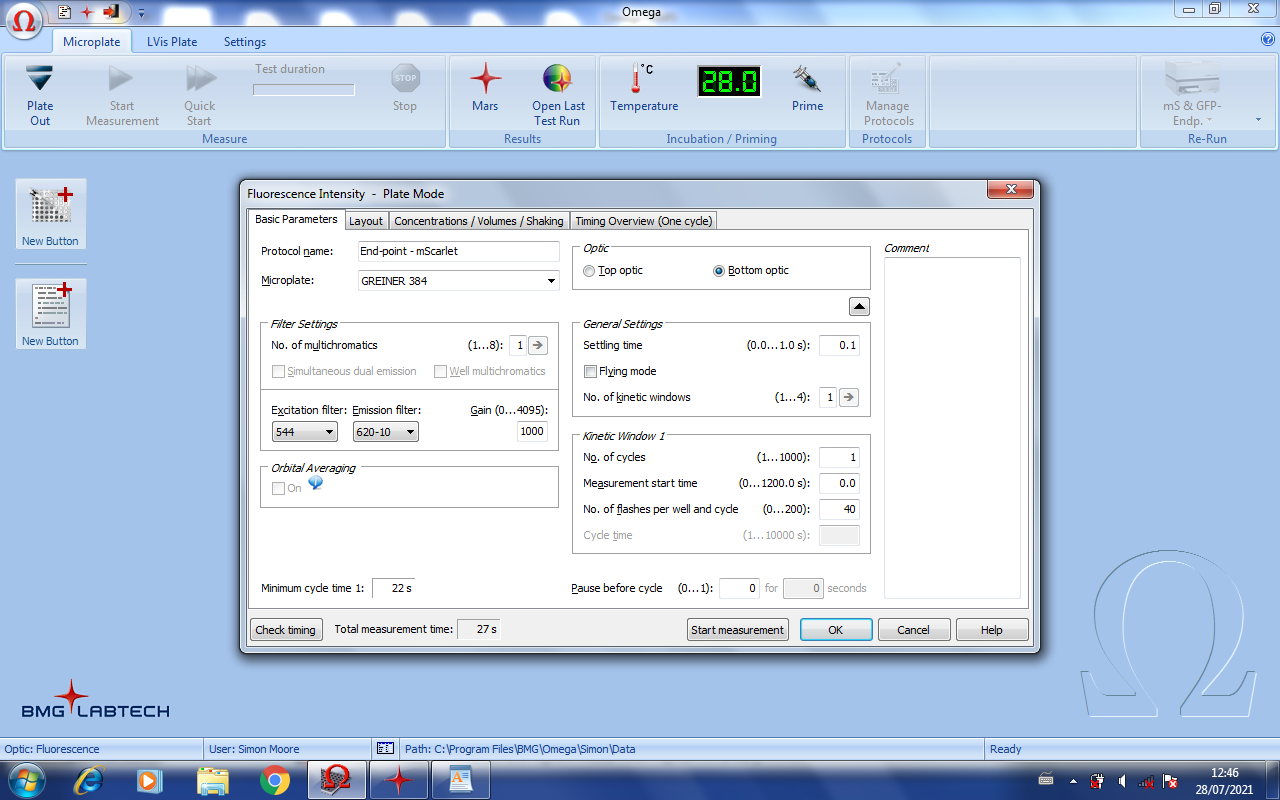


Figure 7. Settings used for an end-point fluorescence measurement.

References:

1. Levine, M.Z. et al. Activation of energy metabolism through growth media reformulation enables a 24-hour workflow for cell-free expression. *ACS Synth Biol*. **9**(10), 2765-2774 (2020).

2. Bindels, D.S. et al. mScarlet: a bright monomeric red fluorescent protein for cellular imaging. *Nature Methods*. **14**(1), 53-56 (2016).
