# Supplementary material for: A High-Yield Streptomyces TX-TL Toolkit for Synthetic Biology and Natural Product Applications
Source: J Vis Exp. Author manuscript; Available in PMC 2023 Aug 10. (PMC7614929; doi:10.3791/63012)
Supplement: Supplementary file S4 [file EMS154240-supplement-Supplementary_file_S4.docx]

**In-gel fluorescence staining of FlAsH-tagged recombinant proteins from TX-TL**

Prepare the following:

**4X SDS-PAGE loading buffer**

200 mM Tris-HCl (pH 6.8)

8% (w/v) sodium dodecyl sulfate

0.4% (w/v) bromophenol blue

40% (v/v) glycerol

**FlAsH-EDT2 dilution**

30 mM FlAsH-EDT_2_ in 50 μL of DMSO

Dilute to 1 mM (10X) in ddH_2_O – slightly insoluble, vortex before pipetting in next steps

**0.5 M TCEP – 5 mL**

**Sample preparation**

1. For a 33 μL cell-free sample, add 1 mL ice-cold acetone
2. Store in freezer for 1 h, or freeze on dry ice
3. Spin down at 16,000 x *g*, 10 min, RT
4. Remove supernatant
5. Wash pellet with 1 mL ice-cold 70% acetone
6. Spin down at 16,000 x *g*, 10 min, RT
7. Remove supernatant and dry pellet
8. Add the following:
9. 22 μL ddH_2_O
10. 10 μL 4X SDS-PAGE loading buffer
11. 4 μL 0.5 M TCEP
12. Boil for 5 minutes
13. Add 4 μL 1 mM FlAsH-EDT
14. Incubate for 15-30 min at RT
15. Centrifuge for 10 min, 16,000 x *g*
16. Add 5 μL of supernatant onto SDS-PAGE following manufacturer guidelines. Avoid overloading, as this leads to smearing of the bands. Optional: run a fluorescent protein ladder (Biorad - Precision Plus Protein™ WesternC™ Blotting Standards) or purified FlAsH-tagged standard protein in parallel
17. Visualise unstained gels on an ultraviolet light-box or by using a camera and Xe-light source with appropriate filters for FlAsH fluorescence (excitation at 480 nm, with emission at 535 nm)
18. Optional - Gels can be stained with routine Coomasie Blue staining, or used for Western blotting analysis
