# Supplementary material for: A High-Yield Streptomyces TX-TL Toolkit for Synthetic Biology and Natural Product Applications
Source: J Vis Exp. Author manuscript; Available in PMC 2023 Aug 10. (PMC7614929; doi:10.3791/63012)
Supplement: Supplementary file S5 [file EMS154240-supplement-Supplementary_file_S5.docx]

**Semi-continuous reaction, metabolite clean-up and HPLC-MS analysis**

TX-TL reactions were prepared as two components (A and B) in a semi-continuous reaction as follows:

1. Component A - 100 μL of standard TX-TL reaction, in the absence of PEG, was injected into a Thermo Scientific Pierce 3.5K MWCO 96-well microdialysis device
2. Component B - 1.5 mL SMM solution with 1 mg/mL carbenicillin was added to a 2.5 mL tube. Note: carbenicillin is supplemented to minimise microbial contamination during the reaction time course.
3. The microdialysis cassette was placed inside the 2.5 mL tube and incubated at 30°C for 24 hrs with shaking (1000 rpm).
4. Acidy samples with 1% (v/v) HCl.
5. Centrifuge at 16,000 x *g* for 30 min at room temperature.
6. Pre-equilibrate a Sep-Pak C-18 (50 mg sorbent) solid-phase extraction cartridge (Waters) with 20 mL of acetonitrile and then 20 mL 1% (v/v) HCl.
7. Load acidified supernatant onto C-18 cartridge, apply pressure with a syringe or manual air pump.
8. Wash with 10 mL of 10% (v/v) ethanol.
9. Elute metabolites with 2 mL of 50% (v/v) ethanol
10. All solutions were acidified with 1% (v/v) HCl.
11. Dry samples using an vacuum centrigue for 30 min.
12. Dissolve samples in 10 μL MeOH and add 90 μL 1% HCl.
13. Centrifuge at 18,000 x *g* for 30 min at room temperature.
14. Load 1 μL of supernatant onto available LC-MS for analysis. Further details of the HPLC-MS set-up are provided below.

**Equipment and HPLC-MS settings used for uroporphyrin III detection**

Reaction products were analysed with an Agilent 1290 Infinity system with an online diode array detector in combination with a Bruker 6500 quadruple time-of-flight (Q-ToF) mass spectrometer. An Agilent Extend-C18 2.1 x 50mm (1.8 μm particle size) column was used at a temperature of 40°C with a buffer flow rate of 0.5 mL/min. LC was performed with a gradient of buffer A (0.1% formic acid in water) and buffer B (0.1% formic acid in acetonitrile). Separation was achieved using 2% buffer B for 0.6 min, followed by a linear gradient to 100% buffer B from 0.6 - 4.6 min, which was held at 100% buffer B from 4.6 - 5.6 min followed by a return to 2% buffer B from 5.6 - 6.6 min, along with 1 min post run. Spectra were recorded between a mass range of 50-1700 *m/z* at a rate of 10 spectra per second in positive polarity.
