# Supplementary material for: A High-Yield Streptomyces TX-TL Toolkit for Synthetic Biology and Natural Product Applications
Source: J Vis Exp. Author manuscript; Available in PMC 2023 Aug 10. (PMC7614929; doi:10.3791/63012)
Supplement: Table S1 [file EMS154240-supplement-Table_S1.pdf]

Chemical	Company	Catalog Number
RTS amino acid sampler	5 Prime	2401530
Ammonium chloride (98%)	Fluorochem	44722
Magnesium chloride (98%)	Fluorochem	494356
Sodium chloride (99%)	Fluorochem	94554
D-(+)-glucose (contact supplier for purity)	Melford	G32040
HEPES Free Acid (contact supplier for purity)	Melford	B2001
Carbenicillin (contact supplier for purity)	Melford	C46000-25.0
DTT (contact supplier for purity)	Melford	MB1015
Yeast Extract	Melford	Y1333
FlAsH-EDT_2 (contact supplier for purity)	Santa Cruz Biotech	sc-363644
Potassium glutamate (>99%)	Sigma	G1149
L-glutamic acid hemimagnesium salt tetrahydrate (>98%)	Sigma	49605
PEG-6000	Sigma	807491
3-PGA (>93%)	Sigma	P8877
Glucose-6-phosphate (>98%)	Sigma	G7879
Poly(vinyl sulfate) potassium salt	Sigma	271969
Malt extract	Sigma	70167-500G
DFHBI (≥98% - HPLC)	Sigma	SML1627
Supelclean™ LC-18 SPE C-18 SPE column (1 g)	Sigma	505471
ATP, CTP, UTP, GTP (100 mM solution, >99%)	ThermoFisher	R0481
384 Well Black/Clear Bottom Plate	ThermoFisher	10692202
Pierce™ 96-well Microdialysis Plate, 10K MWCO	ThermoFisher	88260
2.5L UltraYield Flask	Thomson	931136-B
