# Supplementary material for: A High-Yield Streptomyces TX-TL Toolkit for Synthetic Biology and Natural Product Applications
Source: J Vis Exp. Author manuscript; Available in PMC 2023 Aug 10. (PMC7614929; doi:10.3791/63012)
Supplement: Table S2 [file EMS154240-supplement-Table_S2.pdf]

**Description**	**AddGene No.**
pTU1-A-SP44-PET-*mScarlet-I*	163756
pTU1-A-SP44-PET-*sfGFP*	163757
pTU1-A-SP44-PET-*mVenus-I*	163763
pTU1-B-SP44s-PET-*gus*	172430
pSFC-1A-RFP (StrepFlex, accepts four-five EcoFlex Level 0 plasmids)	172431
pSFC-2A-RFP (StrepFlex, accepts four EcoFlex Level 1 plasmids)	172432
